# Salivary and Microbiome Biomarkers in Periodontitis: Advances in Diagnosis and Therapy—A Narrative Review

**DOI:** 10.3390/medicina61101818

**Published:** 2025-10-11

**Authors:** Casandra-Maria Radu, Carmen Corina Radu, Dana Carmen Zaha

**Affiliations:** 1 Doctoral School of Biological and Biomedical Sciences, University of Oradea, 1 University Street, 410087 Oradea, Romania; dzaha@uoradea.ro; 2 Department of Forensic Medicine, George Emil Palade University of Medicine, Pharmacy, Science, and Technology of Targu Mures, 38 Gheorghe Marinescu Street, 540139 Targu Mures, Romania; carmen.radu@umfst.ro; 3 Department of Preclinical Disciplines, Faculty of Medicine and Pharmacy, University of Oradea, 1 December Square, 410028 Oradea, Romania

**Keywords:** periodontitis, salivary biomarkers, microbiome, precision diagnostics, biomarker-guided therapy, narrative review

## Abstract

*Background and Objectives:* Periodontitis is a common chronic inflammatory disease and a leading cause of tooth loss worldwide. Traditional diagnostic methods, such as probing and radiographic assessment, are retrospective and fail to detect ongoing disease activity. In recent years, salivary biomarkers and oral microbiome profiling have emerged as promising tools for earlier detection and precision-based management. The aim of this review is to synthesize current evidence on salivary and microbiome-derived biomarkers in periodontitis and to evaluate their translational potential in diagnostics and therapy. *Materials and Methods:* A narrative review was performed using PubMed, Scopus, and Web of Science to identify studies published between 2020 and 2025. Search terms included periodontitis, salivary biomarkers, oral microbiome, dysbiosis, and precision therapy. Priority was given to systematic reviews, meta-analyses, and translational studies that addressed diagnostic or therapeutic applications. Eligible publications included English-language original studies and reviews reporting on the diagnostic or therapeutic relevance of salivary or microbiome biomarkers in periodontitis. *Results:* Salivary biomarkers such as cytokines, matrix metalloproteinases (MMPs), oxidative stress markers, microRNAs, and extracellular vesicles (EVs) show consistent associations with disease activity and treatment outcomes. Oral microbiome studies reveal that both classical pathogens and community-level dysbiosis contribute to disease risk. Translational advances include chairside immunoassays, biosensors, lab-on-a-chip devices, and artificial intelligence (AI)-driven analyses. Biomarker-guided therapies—such as microbiome modulation, natural bioactive compounds, host-response modulation, and smart biomaterials—are being evaluated with increasing frequency in translational studies. *Conclusions:* By integrating salivary and microbiome biomarkers with novel diagnostic technologies and emerging therapies, this review complements existing systematic evidence and offers a translational roadmap toward precision periodontology.

## 1. Introduction

Periodontitis is a chronic, multifactorial inflammatory disease characterized by the progressive destruction of the periodontal ligament, alveolar bone, and surrounding connective tissues, ultimately resulting in tooth mobility and, in advanced stages, tooth loss if untreated [1]. The disease originates from a dysbiotic interaction between the oral microbiota and host immune responses, where microbial biofilms trigger sustained inflammation and tissue breakdown. Globally, periodontitis is among the most prevalent oral disorders and represents a substantial burden on both oral and general health. According to the Global Burden of Disease Study, severe periodontitis ranks as the sixth most common disease worldwide, affecting over 700 million individuals [2,3]. Estimates suggest that up to 50% of adults present some degree of periodontal involvement, with severe forms impacting approximately 10–15% of the population [4].

Beyond tooth loss, periodontitis has been associated with systemic conditions such as cardiovascular disease, diabetes mellitus, chronic kidney disease, rheumatoid arthritis, respiratory infections, and adverse pregnancy outcomes [5,6]. This bidirectional relationship underscores the importance of early diagnosis and effective management, not only for preserving oral health but also to improve systemic health outcomes.

Despite therapeutic advances, conventional diagnostic methods remain retrospective and provide limited insight into current or future disease activity, making them unsuitable for early or subclinical detection [7]. These limitations highlight the need for diagnostic tools that are non-invasive, reproducible, and capable of identifying active disease processes before irreversible damage occurs. In this context, host- and microbe-derived biomarkers have gained increasing attention. Saliva is particularly promising because it can be collected non-invasively and reflects both local and systemic conditions [8,9]. Parallel to this, oral microbiome research has moved beyond the pathogen-centric “red complex” to the broader concept of dysbiosis, in which community-level imbalances drive inflammation and tissue destruction. The implications of dysbiosis are discussed further in Section 3 [10,11].

Several systematic reviews and meta-analyses have rigorously evaluated individual salivary biomarkers, such as Toll-like receptors and matrix metalloproteinases, or synthesized evidence for biomarker-based diagnostics [12,13]. While these provide valuable accuracy estimates, their scope remains narrow. This review differs by adopting a narrative and translational perspective, integrating salivary and microbiome-derived biomarkers with recent advances in diagnostic technologies and biomarker-guided therapies [14].

These discoveries are now being integrated into novel diagnostic platforms such as chairside tests, lab-on-a-chip devices, and biosensors [15,16]. AI-assisted analyses of salivary and microbiome datasets further enhance the ability to identify patterns predictive of disease progression [17]. Importantly, several molecular diagnostic kits for detecting major periodontal pathogens in gingival crevicular fluid (GCF) are already commercially available, bridging the gap between experimental research and clinical application. Recent exploratory work with SmartGel OV, a kit was employed to detect 11 key periodontal pathogens in GCF, complementing host-response biomarker analysis and providing a more comprehensive picture of periodontal status [9,11]. This illustrates how integrated diagnostic approaches can support both patient stratification and the development of personalized therapeutic strategies.

Knowledge Gap and Aim of the Review: Several systematic reviews and meta-analyses have examined isolated biomarkers, such as MMP-8 or Toll-like receptors, primarily focusing on their diagnostic accuracy. However, these studies remain fragmented, rarely integrating salivary and microbiome biomarkers within a translational framework or linking them to emerging diagnostic technologies and therapeutic innovations. This creates a gap in the literature regarding how biomarker discoveries can be translated into precision periodontology. Therefore, the aim of this narrative review is to synthesize recent evidence on salivary and microbiome biomarkers, highlight their integration with novel diagnostic platforms, and critically discuss their role in guiding personalized therapeutic strategies.

## 2. Materials and Methods

This review was conducted as a narrative synthesis of the literature. Although narrative reviews do not follow a standardized framework such as PRISMA, we sought to ensure transparency and reproducibility by clearly describing our search process and inclusion criteria. Relevant studies were identified through PubMed, Scopus, and Web of Science between January 2020 to July 2025 using combinations of the terms “periodontitis”, “salivary biomarkers”, “oral microbiome”, “dysbiosis”, and “precision therapy”. Priority was given to systematic reviews, meta-analyses, and translational studies that explicitly evaluated diagnostic or therapeutic applications of salivary and microbiome biomarkers.

The initial search in PubMed using “periodontitis” and “salivary biomarkers” yielded 160 results. After screening titles and abstracts, duplicate entries were removed and additional filters were applied (“oral microbiome”, “dysbiosis”). Similar searches of Scopus and Web of Science were performed. Following the removal of duplicates and application of inclusion/exclusion criteria, a total of 100 articles were selected for full-text review and analysis.

Eligible publications included English-language systematic reviews, meta-analyses, original clinical or translational studies, and narrative reviews reporting on the diagnostic or therapeutic relevance of salivary or microbiome biomarkers in periodontitis. Exclusion criteria were: (1) studies limited to in vitro or animal models without translational significance; (2) publications not directly focused on periodontal disease; and (3) papers without accessible full text.

Because narrative reviews inherently involve subjective interpretation, we attempted to minimize bias by having two authors independently screen and summarize evidence, with disagreements resolved through consensus. The emphasis was placed on reproducibility across populations, validation status of biomarkers, and clinical translation potential, rather than on exhaustive coverage.

## 3. Salivary Biomarkers in Periodontitis

### 3.1. Saliva as a Diagnostic Fluid

Saliva has emerged as one of the most promising biofluids for periodontal diagnostics due to its non-invasive, simple, and cost-effective collection. Unlike GCF, which requires site-specific sampling, saliva provides a composite reflection of the oral cavity and contains host- and microbe-derived molecules, extracellular vesicles, nucleic acids, and metabolites [18,19]. Its composition makes it highly suitable for detecting inflammatory mediators and microbial byproducts associated with periodontal disease. In addition, salivary sampling can be easily standardized for large-scale screenings, making it particularly relevant for both clinical and public health applications [20,21].

### 3.2. Inflammatory Cytokines

Pro-inflammatory cytokines represent one of the most extensively studied groups of salivary biomarkers in periodontitis. Interleukin-1β (IL-1β) is consistently elevated in patients with active periodontal disease and correlates strongly with pocket depth, attachment loss, and bleeding on probing [22]. Elevated salivary levels of tumor necrosis factor-alpha (TNF-α) and interleukin-6 (IL-6) have similarly been associated with disease severity and progression [23,24,25]. These cytokines are central mediators of host response, driving leukocyte recruitment, osteoclast activation, and connective tissue breakdown. Emerging studies also suggest that anti-inflammatory cytokines such as IL-10 and IL-4 may serve as potential biomarkers for monitoring treatment response and resolution of inflammation [26,27,28].

### 3.3. MMPs

MMPs, particularly MMP-8 (neutrophil collagenase) and MMP-9 (gelatinase B), are critical enzymes in the degradation of extracellular matrix components [9,29,30]. Elevated levels of salivary MMP-8 have been repeatedly linked with active periodontal tissue destruction. The clinical translation of MMP-8 into point-of-care assays is further discussed in Section 4 [31]. MMP-9, while less specific, is also commonly elevated in periodontitis and correlates with disease activity. In combination with cytokines, MMP levels can provide complementary information on the inflammatory and tissue-destructive phases of periodontal disease [32,33].

### 3.4. Oxidative Stress Markers

Periodontitis is characterized not only by inflammation but also by oxidative stress. Salivary biomarkers of oxidative damage, including 8-hydroxydeoxyguanosine (8-OHdG), malondialdehyde (MDA), and advanced oxidation protein products (AOPPs), have been associated with periodontal disease severity [34,35]. Total antioxidant capacity (TAC) is often reduced in periodontitis, reflecting disrupted oxidant–antioxidant balance [36]. These markers add an additional layer of diagnostic information by capturing the oxidative stress component of periodontal pathogenesis [37,38].

### 3.5. Emerging and Novel Biomarkers

Beyond cytokines and proteolytic enzymes, several emerging salivary biomarkers are gaining attention. MicroRNAs (miRNAs), such as miR-146a and miR-155, have been implicated in regulating immune and inflammatory pathways and may serve as sensitive molecular indicators of disease activity [39,40]. EVs, which carry proteins, nucleic acids, and lipids, are another promising frontier, with recent studies indicating that EV cargo can differentiate between health and disease [41,42,43,44]. Additionally, metabolites detected through salivary metabolomics—such as short-chain fatty acids (SCFAs) and amino acid derivatives—offer insights into host–microbe interactions and may support the development of metabolic biomarker panels [45,46].

### 3.6. Translation into Clinical Diagnostics

The transition from research to clinical practice is exemplified by the development of salivary diagnostic kits and biosensor technologies [12,47,48]. Multiplex platforms capable of simultaneously quantifying multiple cytokines and enzymes are under development, and lab-on-a-chip biosensors offer the potential for point-of-care, real-time diagnostics [8]. Importantly, integration of salivary biomarkers with microbiome profiling and AI-based analysis may enable comprehensive diagnostic platforms that surpass the predictive value of traditional clinical indices [49].

## 4. Oral Microbiome and Biomarkers in Periodontitis

### 4.1. Dysbiosis as a Central Concept

The oral microbiome is a highly diverse ecosystem, comprising more than 700 bacterial species, as well as fungi, viruses, and archaea [50,51]. Periodontal health is maintained by a symbiotic microbial community that coexists with host defenses. In contrast, periodontitis is associated with a shift toward dysbiosis, in which microbial diversity increases and pro-inflammatory species gain ecological advantage [52,53]. This dysbiotic state contributes to persistent inflammation, tissue destruction, and ultimately clinical disease progression. Importantly, dysbiosis is not driven by a single pathogen but results from complex ecological imbalances that alter microbial community structure and function [54].

### 4.2. Classical Pathogens and the “Red Complex”

Historically, periodontal pathogenesis has been attributed to a group of anaerobic Gram-negative bacteria collectively known as the “red complex”: *Porphyromonas gingivalis*, *Tannerella forsythia*, and *Treponema denticola* [55]. These species are strongly associated with clinical attachment loss and deep periodontal pockets and have been widely used as microbial markers of disease. Additional taxa such as *Aggregatibacter actinomycetemcomitans* (particularly in aggressive forms of periodontitis) have also been implicated [56,57]. While valuable, this pathogen-centric model has been increasingly recognized as overly simplistic, as it fails to explain disease heterogeneity and progression in the absence of these species.

### 4.3. Microbial Signatures Beyond the “Red Complex”

High-throughput sequencing has expanded the list of taxa associated with periodontitis, identifying new microbial signatures that distinguish health, gingivitis, and periodontitis. Species such as *Filifactor alocis*, *Prevotella intermedia*, *Fusobacterium nucleatum*, and *Peptostreptococcus stomatis* have been linked with disease activity [58,59]. Moreover, shifts in the relative abundance of commensal organisms—such as reductions in *Streptococcus sanguinis* and *Streptococcus gordonii*—also contribute to dysbiosis [60,61,62]. These findings support the concept of a “polymicrobial synergy and dysbiosis” model, where disease arises not from a single organism but from community-level interactions and host immune modulation [63].

### 4.4. Functional and Metagenomic Biomarkers

Recent advances in shotgun metagenomics and meta transcriptomics have revealed that functional changes in the microbiome may be more informative than the presence of specific taxa [14,64]. Periodontitis-associated communities are enriched in genes related to proteolysis, iron acquisition, lipopolysaccharide biosynthesis, and SCFA metabolism [65]. Similarly, transcriptomic analyses indicate that microbial virulence gene expression, rather than mere presence, correlates more closely with disease activity [46]. These functional biomarkers provide mechanistic insights into how microbial dysbiosis drives inflammation and tissue destruction, while also offering novel targets for diagnostic and therapeutic intervention [66].

### 4.5. Viral and Fungal Contributions

Although less studied than bacteria, viruses and fungi may also serve as relevant biomarkers of dysbiosis [64]. Periodontal pockets harbor diverse viral communities, including bacteriophages that modulate bacterial populations and host interactions. Candida species, particularly *Candida albicans*, have been identified more frequently in diseased sites and may synergize with bacteria to exacerbate inflammation [67]. Understanding the role of non-bacterial members of the oral microbiome could further refine biomarker-based approaches to periodontal diagnostics [68].

### 4.6. Translational Applications of Microbiome Biomarkers

Microbiome biomarkers are increasingly being incorporated into diagnostic platforms. Commercial test kits capable of detecting panels of periodontal pathogens in subgingival plaque or gingival crevicular fluid are already in use in research and select clinical contexts. These tests, including those applied in exploratory studies such as the SmartGel OV project, can detect up to 11 periodontal pathogens simultaneously, providing clinicians with a molecular profile of the microbial community [69]. Coupled with salivary biomarkers, these tools offer a more comprehensive assessment of disease risk and activity. Looking forward, AI-driven integration of microbial, host, and clinical data may allow real-time risk prediction and patient-specific treatment planning, marking a substantial step toward precision periodontology [70,71].

A summary of the principal salivary and microbiome biomarkers associated with periodontitis, their biological roles, and diagnostic relevance is presented in Table 1.

**Figure 1:**
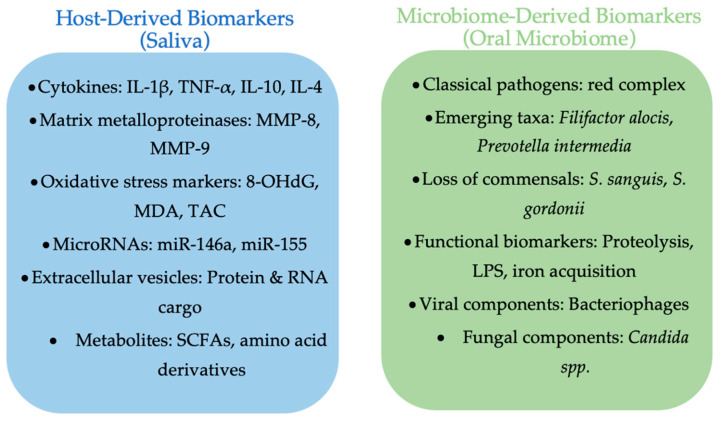
Comparative overview of salivary and oral microbiome biomarkers in periodontitis. Salivary biomarkers include cytokines, proteolytic enzymes, oxidative stress markers, microRNAs, extracellular vesicles, and metabolites. Microbiome biomarkers encompass classical red complex species, emerging taxa, loss of commensals, functional gene shifts, as well as viral and fungal components. Together, these biomarker categories provide complementary insights into periodontal disease activity and progression (made with an AI-assisted design manager, accessed on 15 July 2025).

A comparative overview of salivary and microbiome biomarkers in periodontitis is presented in Figure 1, highlighting the complementary insights provided by host- and microbe-derived indicators of disease activity.

## 5. Novel Diagnostic Tools for Periodontitis

### 5.1. Limitations of Conventional Methods

Traditional periodontal diagnostics rely on PD, CAL, BOP, and radiographic evaluation [81]. While these parameters remain the clinical gold standard, they are retrospective in nature and provide little insight into ongoing disease activity or future risk. This limitation has driven the development of novel diagnostic technologies that integrate host- and microbe-derived biomarkers with advanced analytical platforms [82].

### 5.2. Chairside Tests and Immunoassays

Point-of-care assays have emerged as practical tools for rapid detection of periodontal inflammation. Chairside kits such as PerioSafe^®^ and ImplantSafe^®^ (Dentognostics GmgH, Solingen, Germany), which detect active MMP-8 in saliva or GCF, allow clinicians to identify active tissue breakdown within minutes [83,84]. These tests are easy to use, cost-effective, and compatible with routine dental visits, making them among the most clinically ready biomarker-based diagnostic tools. However, their specificity and sensitivity still require validation in diverse populations and longitudinal studies [47,72,85].

### 5.3. Biosensors and Lab-on-a-Chip Devices

Advances in microfluidics and nanotechnology have enabled the development of biosensors and lab-on-a-chip devices capable of detecting multiple biomarkers simultaneously [8]. These platforms can integrate immunoassays, DNA hybridization, and electrochemical detection to measure cytokines, enzymes, and microbial DNA in small saliva or GCF samples. Their portability and rapid turnaround times make them highly suitable for chairside application and population-level screening. Recent prototypes have demonstrated high sensitivity and specificity for MMP-8, IL-6, and microbial DNA, suggesting strong potential for clinical translation [15,86].

### 5.4. High-Throughput Molecular Diagnostics

Molecular diagnostic assays based on polymerase chain reaction (PCR) and quantitative PCR (qPCR) remain widely used for detecting periodontal pathogens. Commercial kits capable of profiling multiple species simultaneously represent a bridge between microbiome research and practical application [40,87]. More recently, next-generation sequencing (NGS) approaches have been explored for comprehensive profiling of oral microbial communities. Although NGS remains expensive and time-consuming, its ability to identify microbial and functional signatures with high resolution offers unique potential for future diagnostic frameworks [88].

### 5.5. AI and Data Integration

AI is increasingly being applied to integrate complex datasets combining salivary biomarkers, microbiome profiles, and clinical parameters [89]. Machine learning models have shown promising results in predicting disease status, progression, and response to therapy. AI-assisted image analysis of radiographs and periodontal charts also offers enhanced diagnostic accuracy and reproducibility [90]. The integration of AI with biomarker-based diagnostics may ultimately enable personalized risk prediction and treatment planning, moving periodontal care closer to precision medicine [89,91,92,93].

### 5.6. Challenges for Clinical Translation

While the potential of novel diagnostic technologies is considerable, several barriers remain. Cost-effectiveness, ease of use, and standardization across laboratories are critical for adoption in daily practice. Regulatory approval and clinical validation in large, diverse populations will be necessary before widespread implementation. Furthermore, integration of these tools into existing dental workflows will require both clinician training and patient acceptance [72,94]. Addressing these challenges will be essential for moving from proof-of-concept prototypes to routine clinical use. The main categories of novel diagnostic platforms, their applications, advantages, and limitations are summarized in Table 2.

## 6. Biomarker-Guided Therapeutic Innovations

### 6.1. From Conventional to Precision Periodontal Therapy

Traditional periodontal therapy has relied on mechanical debridement, adjunctive antimicrobials, and surgical approaches to control microbial biofilms and inflammation. While effective in many cases, these treatments are often applied uniformly across patients, without consideration of individual host–microbe profiles. Biomarker-driven strategies represent a paradigm shift, enabling clinicians to stratify patients according to their inflammatory and microbial status and to tailor therapy accordingly [95].

### 6.2. Microbiome Modulation: Probiotics, Prebiotics, and Postbiotics

Microbiome modulation is emerging as a complementary therapeutic strategy. Probiotics such as *Lactobacillus reuteri* have been shown to reduce gingival inflammation and modulate microbial balance in patients with periodontitis [96,97]. Prebiotics—substrates that selectively promote the growth of beneficial bacteria—offer another means of restoring microbial homeostasis. More recently, postbiotics (non-viable microbial products with bioactive properties) have gained attention for their ability to modulate immune responses and reduce inflammation [98]. The identification of specific microbial biomarkers enables targeted application of these therapies, maximizing their effectiveness [97].

### 6.3. Natural Bioactive Compounds and Essential Oils (EOs)

Natural compounds, particularly EOs, have demonstrated antimicrobial, anti-inflammatory, and antioxidant effects relevant to periodontitis [99,100,101]. Compounds such as carvacrol, thymol, and linalool have been shown to inhibit key periodontal pathogens and disrupt biofilms while modulating host immune responses [102]. Salivary and microbiome biomarkers can be used to monitor treatment efficacy, offering an objective framework for integrating natural bioactives as adjunctive therapies. Given the global challenge of antibiotic resistance, such biomarker-guided natural interventions represent a promising alternative in periodontal care [103,104].

### 6.4. Host-Response Modulation Therapies

In addition to targeting microbial dysbiosis, host-modulating therapies are increasingly being explored. Sub-antimicrobial dose doxycycline (SDD) is already used to inhibit MMP activity and reduce collagen breakdown [29,105]. Novel agents aimed at blocking pro-inflammatory cytokines or enhancing anti-inflammatory pathways are in development. The measurement of salivary cytokines and MMPs provides a means of tailoring host-modulation therapies to individual patients, optimizing their efficacy and safety [9].

### 6.5. Smart Biomaterials and Local Drug Delivery

Advances in biomaterials science have enabled the design of smart drug delivery systems capable of releasing therapeutic agents directly into periodontal pockets [89]. Hydrogels, nanofibers, and biodegradable microspheres have been engineered to provide controlled, sustained release of antimicrobials, anti-inflammatory drugs, or bioactive molecules [106,107]. By coupling biomarker detection (e.g., pathogen panels, cytokine levels) with localized release of antimicrobial or anti-inflammatory agents, such technologies exemplify the future of precision periodontal therapy [9,108].

### 6.6. Integration into Clinical Practice

Although many biomarker-guided therapies remain at the experimental or early clinical stage, their translational potential is substantial [48,109]. Personalized interventions guided by biomarker profiles could improve treatment outcomes, reduce unnecessary antibiotic use, and minimize disease recurrence [110,111,112]. However, clinical adoption will require robust validation through randomized controlled trials, regulatory approval, and demonstration of cost-effectiveness. Interdisciplinary collaboration between periodontists, microbiologists, biomaterials scientists, and data specialists will be crucial for bringing these innovations into routine practice [109,113].

**Figure 2:**
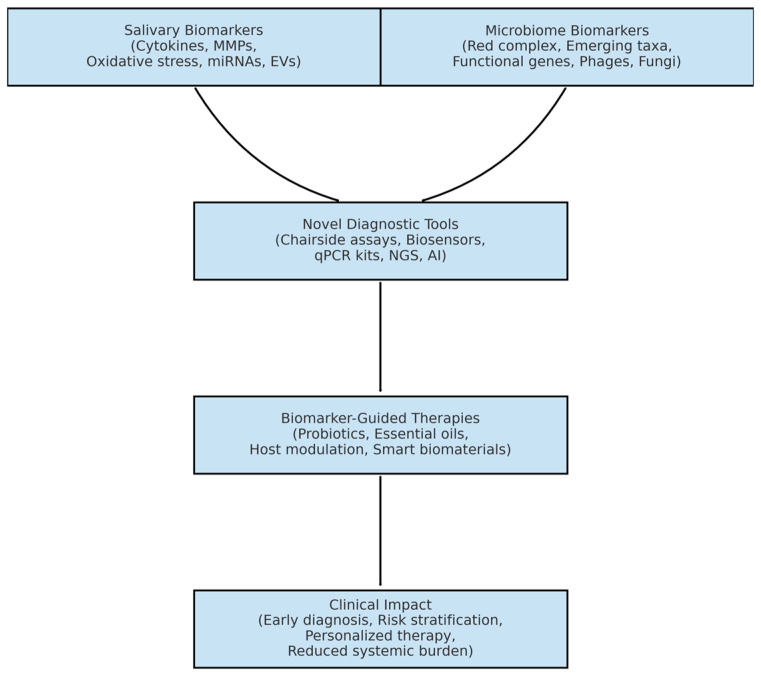
Conceptual framework illustrating the role of salivary and oral microbiome biomarkers in periodontitis. Biomarker discovery informs the development of novel diagnostic platforms, which in turn guide biomarker-based therapeutic innovations, enabling precision diagnostics, personalized treatment, and improved clinical outcomes (made with an AI-assisted design manager, accessed on 15 July 2025).

Biomarker discovery informs the development of novel diagnostic platforms, which in turn guide biomarker-based therapeutic innovations. Together, these advances enable a transition from conventional symptom-based management toward precision diagnostics, personalized treatment, and improved clinical outcomes, as illustrated in Figure 2.

## 7. Discussion

Recent advances in salivary and microbiome biomarkers highlight a shift from symptom-based diagnosis of periodontitis toward molecularly informed strategies. Saliva offers clear advantages as a diagnostic fluid: it is easily accessible, non-invasive, and reflects both local periodontal status and systemic conditions. Biomarkers such as cytokines (IL-1β, IL-6, TNF-α), MMPs (particularly MMP-8), and oxidative stress indicators have consistently demonstrated associations with disease severity and treatment response. Similarly, oral microbiome studies have moved beyond the “red complex” pathogens to reveal community-level dysbiosis as a driver of inflammation and tissue destruction.

Despite this progress, important challenges remain. Biomarker studies vary widely in their design, assay platforms, and populations, making cross-study comparisons difficult and limiting the definition of universal diagnostic thresholds. Many associations remain correlational, and the causal role of microbial shifts has not been fully established. In addition, reproducibility issues, regulatory approval, and the lack of cost-effectiveness data hinder large-scale clinical adoption.

Beyond the general challenges of biomarker validation, the study of oral microbiome biomarkers faces significant methodological variability. Differences in sampling sites (e.g., saliva, subgingival plaque, gingival crevicular fluid), sequencing methodologies (16S rRNA vs. shotgun metagenomics), and bioinformatic pipelines can lead to inconsistent findings and reduced reproducibility across studies. Recent research in oral potentially malignant disorders and oral squamous cell carcinoma has similarly highlighted these limitations, underscoring the need for methodological rigor and standardized protocols when translating microbiome data into clinical settings [114].

Translational progress, however, is evident. Chairside immunoassays, biosensors, and lab-on-a-chip devices now enable the rapid detection of host and microbial markers in real time. AI strengthens diagnostic accuracy by integrating complex datasets and identifying predictive patterns. The availability of commercial kits for detecting multiple periodontal pathogens in gingival crevicular fluid demonstrates how molecular diagnostics can complement clinical indices. Multiplex approaches that combine salivary and microbial markers may allow for improved patient stratification and more personalized interventions.

Looking forward, several priorities are clear. Standardization of assays and validation in large, diverse cohorts are essential to ensure reproducibility and clinical reliability. Longitudinal studies are needed to confirm whether biomarker profiles can truly predict progression and treatment response. Integration of biomarker data with imaging, clinical indices, and behavioral factors through AI-driven platforms holds promise for risk prediction models suitable for routine practice. On the therapeutic side, biomarker-guided strategies—such as microbiome modulation, natural bioactive compounds, host-response modulators, and smart biomaterials—require robust clinical trials to demonstrate safety, efficacy, and cost-effectiveness.

Current evidence suggests that not all biomarkers are equally validated. Host-derived markers such as IL-1β and active MMP-8 show the strongest reproducibility across diverse populations and are closest to clinical translation, as reflected in the availability of chairside assays. IL-6, TNF-α, and oxidative stress markers also demonstrate consistent associations, but assay variability and lack of standardized cut-off values remain barriers. In contrast, extracellular vesicles, microRNAs, and salivary metabolites remain exploratory, with evidence limited to small pilot studies and preclinical investigations. These emerging biomarkers highlight exciting directions but require large, multicenter studies before they can be considered for routine clinical use. While cytokines and MMPs (particularly IL-1β and active MMP-8) are supported by relatively robust validation and even chairside assays, other categories of salivary biomarkers—including extracellular vesicles, microRNAs, and salivary metabolites—remain largely exploratory. Most of the current evidence comes from single-center studies with small sample sizes, heterogeneous methodologies, and limited reproducibility. To avoid overinterpretation, it is essential to emphasize that these biomarkers require validation in large-scale, multicenter cohorts using standardized protocols before they can be translated into clinical diagnostics. Establishing such methodological and population-level robustness will be critical for distinguishing truly reliable markers from those that may reflect context-specific findings [25].

Current evidence shows that not all biomarkers are equally validated. IL-1β and active MMP-8 are the most reproducible and clinically advanced, supported by chairside assays, while IL-6, TNF-α, and oxidative stress markers remain promising but limited by assay variability. In contrast, EVs, microRNAs, and salivary metabolites are still exploratory and require validation in large, multicenter studies. The oral microbiome provides valuable insights into dysbiosis, yet challenges in standardization and causality persist.

Future research should prioritize large-scale validation, standardized assay platforms, and integration of biomarker data with imaging and clinical indices through AI-driven models. Robust clinical trials of biomarker-guided therapies—including microbiome modulation, natural bioactive compounds, host-response modulators, and smart biomaterials—are essential to establish clinical utility and cost-effectiveness.

By consolidating these perspectives, the present review delivers a translational synthesis that bridges biomarker discovery, diagnostic innovation, and therapeutic development, providing the foundation for the concluding roadmap toward precision periodontology. As this is a narrative review, selection and interpretation of evidence may reflect a degree of author subjectivity. Unlike systematic reviews, our methodology does not eliminate all risk of bias. Nevertheless, by prioritizing high-quality evidence such as systematic reviews, meta-analyses, and translational studies, we aimed to provide a balanced synthesis while acknowledging this limitation.

## 8. Conclusions

Periodontitis remains a major challenge for both oral and systemic health, and conventional diagnostic methods are limited by their retrospective nature. Advances in salivary and oral microbiome biomarkers provide new opportunities for earlier, non-invasive, and more precise disease management.

Among the available markers, IL-1β and active MMP-8 show the strongest reproducibility across populations and are closest to clinical translation, while microRNAs, EVs, and salivary metabolites remain exploratory and require further validation. Emerging diagnostic technologies—such as chairside immunoassays, biosensors, lab-on-a-chip platforms, and AI-driven analyses—are accelerating the translation of biomarker science into practice. Likewise, biomarker-guided therapies, including microbiome modulation, natural bioactive compounds, host-response modulation, and smart biomaterials, are expanding the horizon of precision periodontology.

This review complements systematic evidence by providing an integrated translational perspective that bridges biomarker discovery with diagnostic innovation and therapeutic development. Future progress will depend on assay standardization, validation across diverse populations, and demonstration of cost-effectiveness. Together, these advances provide a roadmap for transforming periodontal care from reactive treatment toward proactive, precision-based prevention.

The successful integration of biomarker-driven diagnostics and therapies into routine clinical workflows will require close interdisciplinary collaboration. Periodontists, microbiologists, biomaterials scientists, and data specialists must work together to translate laboratory discoveries into clinically applicable, cost-effective solutions. Such cross-disciplinary efforts will be essential to overcome current barriers, ensure methodological rigor, and accelerate the adoption of precision-based strategies in periodontology.

## Figures and Tables

**Table 1:** Key salivary and oral microbiome biomarkers in periodontitis and their clinical relevance.

Biomarker Category	Representative Markers	Role in Periodontitis	Clinical/Diagnostic Relevance	Validation Status	References
Salivary Cytokines	IL-1β, IL-6, TNF-α	Drive inflammatory cascade, recruit immune cells, stimulate osteoclastogenesis	Elevated in saliva of periodontitis patients; correlated with probing depth, attachment loss, and disease activity	consistently reported, but cut-off values variable	[39,43]
Anti-inflammatory Cytokines	IL-10, IL-4	Suppress pro-inflammatory pathways	Potential markers of treatment response and resolution of inflammation	well-validated; reproductible across studies	[24,26]
Matrix Metalloproteinases	MMP-8, MMP-9	Collagen and extracellular matrix degradation	Active MMP-8 chairside assays (PerioSafe^®^, ImplantSafe^®^) validated as diagnostic tools	well-validated; reproductible across studies	[19,48,72]
Oxidative Stress Markers	8-OHdG, MDA, AOPPs, TAC	Reflect imbalance between oxidants and antioxidants; contribute to tissue destruction	Elevated in saliva of diseased patients; correlate with severity and progression	consistently reported, but cut-off values variable	[34,36]
MicroRNAs (miRNAs)	miR-146a, miR-155	Regulate immune and inflammatory gene expression	Emerging molecular biomarkers for early disease detection and monitoring	exploratory; limited validation	[40,73]
EVs	Protein and RNA cargo in salivary EVs	Reflect cellular signaling and inflammation	Discriminate between health and periodontitis in pilot studies	exploratory; limited validation	[41,42,74]
Microbial “Red Complex”	*Porphyromonas gingivalis*, *Tannerella forsythia*, *Treponema denticola*	Strongly associated with attachment loss and deep periodontal pockets	Traditional microbial markers; still included in commercial test kits	well-validated; reproductible across studies	[75,76]
Emerging Bacterial Signatures	*Filifactor alocis*, *Prevotella intermedia*, *Fusobacterium nucleatum*, *Peptostreptococcus stomatis*	Linked with disease progression and dysbiosis	Identified via 16S and metagenomics; add predictive value beyond red complex	well-validated; reproductible across studies	[23,50]
Loss of Commensals	*Streptococcus sanguinis*, *Streptococcus gordonii*	Normally protective, maintain symbiosis	Reduction associated with transition from health to disease	well-validated; reproductible across studies	[77,78]
Functional/Molecular Biomarkers	Genes for proteolysis, LPS biosynthesis, iron acquisition, SCFA metabolism	Reflect microbial functions driving inflammation	Provide mechanistic targets; identified by metagenomics/metatranscriptomics	exploratory; limited validation	[72,79]
Viral and Fungal Components	Bacteriophages, *Candida albicans*	Modulate bacterial communities, synergize with pathogens	Potential adjunct biomarkers of dysbiosis; understudied	exploratory; limited validation	[80]

Abbreviations: 8-OHdG, 8-hydroxydeoxyguanosine; MDA, malondialdehyde.

**Table 2:** Novel diagnostic tools for periodontitis and their clinical applications.

Technology/Platform	Examples	Target Biomarkers	Advantages	Limitations/Challenges
Chairside Immunoassays	PerioSafe^®^, ImplantSafe^®^	Active MMP-8 in saliva/GCF	Rapid (minutes), non-invasive, easy for routine use	Limited to single biomarker; variability in sensitivity/specificity
Biosensors and Lab-on-a-Chip	Microfluidic and electrochemical devices	Cytokines (IL-6, IL-1β), MMPs, microbial DNA	Multiplex detection, small sample volume, real-time analysis	Still mostly experimental; cost and calibration issues
PCR/qPCR	Commercial kits (e.g., 11-pathogen GCF panels, used in SmartGel OV work)	Red complex bacteria, *A. actinomycetemcomitans*, emerging pathogens	High sensitivity and specificity; already available commercially	Requires lab infrastructure; cannot capture functional microbial activity
NGS	16S rRNA sequencing, shotgun metagenomics	Comprehensive microbiome and functional pathways	High-resolution analysis; reveals dysbiosis and functional shifts	Expensive, time-consuming, limited clinical practicality
AI Integration	Machine learning models; AI-assisted radiograph/microbiome analysis	Multi-modal data (saliva, microbiome, clinical indices, imaging)	Enhanced prediction of risk and progression; personalized treatment planning	Requires large datasets; interpretability and standardization challenges

## Data Availability

Information provided in this research are supported by the inserted references.
